# Production of Fungal Mycelia in a Temperate Coniferous Forest Shows Distinct Seasonal Patterns

**DOI:** 10.3390/jof6040190

**Published:** 2020-09-26

**Authors:** Martina Štursová, Petr Kohout, Zander Rainier Human, Petr Baldrian

**Affiliations:** Laboratory of Environmental Microbiology, Institute of Microbiology of the Czech Academy of Sciences, Vídeňská 1083, 14220 Prague, Czech Republic; petr.kohout@biomed.cas.cz (P.K.); zander.human@biomed.cas.cz (Z.R.H.)

**Keywords:** fungal ecology, metabarcoding, mycelial growth, ectomycorrhiza, temperate forest, soil fungi, *Picea abies*

## Abstract

In temperate forests, climate seasonality restricts the photosynthetic activity of primary producers to the warm season from spring to autumn, while the cold season with temperatures below the freezing point represents a period of strongly reduced plant activity. Although soil microorganisms are active all-year-round, their expressions show seasonal patterns. This is especially visible on the ectomycorrhizal fungi, the most abundant guild of fungi in coniferous forests. We quantified the production of fungal mycelia using ingrowth sandbags in the organic layer of soil in temperate coniferous forest and analysed the composition of fungal communities in four consecutive seasons. We show that fungal biomass production is as low as 0.029 µg g^−1^ of sand in December–March, while it reaches 0.122 µg g^−1^ in June–September. The majority of fungi show distinct patterns of seasonal mycelial production, with most ectomycorrhizal fungi colonising ingrowth bags in the spring or summer, while the autumn and winter colonisation was mostly due to moulds. Our results indicate that fungal taxa differ in their seasonal patterns of mycelial production. Although fungal biomass turnover appears all-year-round, its rates are much faster in the period of plant activity than in the cold season.

## 1. Introduction

Fungi play important ecological roles in forests: besides the free-living saprotrophs, diverse fungal groups interact with plants as mycorrhizal symbionts or important pathogens. Especially in the temperate and boreal regions, the research on fungi in forest ecosystems substantially increased our understanding of their ecology [1] and biogeography [2]. Due to their interactions with forest trees, fungi greatly contribute to the soil C balance by affecting both the net primary production (NPP) of forests and carbon (C) mineralisation and sequestration [3].

Fungal mycelia form a substantial portion of belowground microbial biomass [1,4], and fungi may represent an important C sink, since the necromass of many mycorrhizal species contributes to long-term C storage in boreal regions [5]. On the other hand, the decomposition of fungal mycelia [6,7] and the contribution of saprotrophic and mycorrhizal fungi to the decomposition of soil organic matter contribute to ecosystem-level C mineralisation [8,9].

In temperate and boreal forests dominated by trees forming ectomycorrhizal (ECM) associations, most of the fungal biomass is formed by ECM fungi [10,11]. Up to almost 40% of NPP may support the production of ECM mycelia [12]. This is, for example, much more than NPP used to find root production, which was estimated below 20% in *Picea abies* [13] and other ECM coniferous forests [12]. The standing biomass of ECM mycelia in the upper 70 cm of the soil profile may be as high as 480 to 580 g m^−2^ [11]. Due to the rapid turnover of ectomycorrhizal mycelia [14,15], the rate of ECM fungal biomass production is high, reaching up to 180 g C m^−2^ y^−2^ and being considerably higher in coniferous than deciduous stands [12,16].

While the C cycling models and predictions have been improved recently by including the data on fungal biomass production [9,10,15,17], the estimates of biomass production only rarely considered the intra-annual variations and the changes of the ecosystem properties during the year. Temperate and boreal forest ecosystems are, indeed, typical by strong seasonal dynamics responding to differing light, temperature or moisture regimes throughout the year [18], which is reflected in both the above and belowground biota and various processes [15,19,20]. One of the apparently dynamic processes is the photosynthetic production of trees that undergoes seasonal cycles of productivity [21]. The seasonal allocation of tree photosynthates (along with old/storage C) into the roots and rhizosphere [21,22,23] as the most rapid route of C allocation to soil potentially affects a wide range of belowground processes. These include the respiration, decomposition or stabilisation of soil organic matter [10], production and activity of extracellular enzymes [24], as well as community composition turnover [25] or belowground biomass production [26]. All of them are driven, directly or indirectly, through the seasonal production and activity of ECM fungi.

The differences in ECM fungal biomass production are visible through the seasonality of total fungal biomass. Its highest stocks were reported in the early autumn for northern ECM coniferous forests [26,27], while ECM deciduous forests have fungal biomass peaking in the summer [28,29] corresponding to the peak belowground allocation of plant-derived C [21]. To target specifically the mycelial production of ectomycorrhizal fungi, ingrowth of mycelia into bags lacking organic C (such as sand-containing bags) was utilised for quantification in the past [30]. Although temporal trends of mycelial production were never the main objective of such studies, differences in mycelial production were seen when incubating bags in two contrasting seasons [31].

In addition to seasonal changes in fungal biomass production, the changes of fungal community composition across seasons was also occasionally observed in ECM deciduous forests [29,32], with winter typically dominated by saprotrophic species and ectomycorrhizal species increasing in abundance during the growing season. In contrast to that, the fungal community composition in a coniferous *Picea abies* forest did not show significant differences between the summer and winter [19].

Our previous analysis of the microbiome seasonality in the ECM coniferous forest soil indicated that the share of fungi on the total microbial transcription is approximately twice as high in late-summer than in late-winter [19]. Using the rate of ribosomal protein production as a proxy of microbial biomass production [33], the share of bacterial ribosomal proteins on the total transcription was slightly higher in the winter (3760 ± 530 per one million of reads) than in the summer (2760 ± 220), while the share of fungal ribosomal protein in the summer was three times higher than in the winter (363 ± 63 and 113 ± 15, respectively). This indicates the relative reduction of fungal growth in the winter [19]. The decrease of fungal activity in the winter was especially visible in the drastic reduction of the share of ectomycorrhiza-specific fungal transcription in the winter [19], linking this seasonal pattern to plant photosynthetic productivity and changes in C utilisation [34].

However, transcriptome analyses only report the activity at the exact time of sampling, and as such, they do not provide reliable information about fungal productivity and activity across whole seasons. Moreover, the low taxonomic resolution of a transcriptome analysis does not allow to address the seasonality of fungal guilds or individual taxa. In this experiment, we addressed the question of seasonal production of extrametrical ECM fungal mycelia in a mature spruce forest using the incubation of ingrowth bags filled with sand across four consecutive three-month periods representing different seasons and analysed the composition of the fungal community within the bags to track the seasonality of mycelial production of different fungal species across the year. Since many fungi depend on root activity and respond to changing temperatures [35,36], we hypothesised that the rate of mycelial production reflects the C availability for fungi, as determined by the soil temperature and tree productivity, and that it peaks in the summer season. Furthermore, we hypothesised that the share of ectomycorrhizal fungi on mycelial production reflects the seasonal rates of rhizodeposition, which are highest in the late-vegetation season.

## 2. Materials and Methods

### 2.1. Study Site, Experimental Setup and Biomass Quantification

The study area was located at high altitudes (1170–1200 m) of the Bohemian Forest mountain range, Czech Republic (Central Europe; 49°02 N, 13°37 E) and was covered by an unmanaged Norway spruce (*Picea abies*) forest. The mean annual temperature was 5.9 °C, and the mean annual precipitation was 1100 mm. The snow cover lasts from November until April, while precipitation shows little seasonality. The understory was either missing or composed of grasses (*Avenella* and *Calamagrostis*), bilberries (*Vaccinium*) and mosses. The same study area was explored previously to identify the total and active microbial communities and to describe the seasonality of the microbial processes [19,33,34,37].

To estimate the seasonal mycelial production by fungi, we prepared 60 fungal ingrowth bags made of nylon mesh (50-µm mesh size, equilateral triangle shape with a side length of 8 cm) and filled with 50-g acid-washed quartz sand (0.30–2.0 mm, 99.6% SiO_2_; Provodínské písky, Provodín, Czech Republic). This mesh size allows fungi, but not roots, to grow through. Starting in June 2017, each three months, we placed 15 bags in the humic horizon (approx. until 10-cm depth) at random locations spanning 5 ha within the sampled area and retrieved them after three months when a new batch of 15 sandbags was placed at the same locations. We refer to the June–September incubation period as summer, September–December as autumn, December–March as winter and March–June as spring. Along with sandbags, soil was sampled at three defined locations within the experimental area to determine the overall community present. At each location, sixteen soil cores (4-cm diameter) were collected up to the same depth where the bags were placed. Litter and roots were removed, and soil was passed through a 5-mm sterile mesh and mixed to give a composite sample for each location. Samples were kept on ice during transport to the laboratory (within 1 day), frozen and kept at −20 °C until freeze-dried. Temperature was recorded using multiple TMS4 probes (TOMST, Prague, Czech Republic [38]) at a 5-cm depth with a 15-min interval.

### 2.2. Biomass Quantification

Fungal biomass in bags was estimated based on the quantification of the ergosterol content [39]. Total ergosterol was extracted and analysed as described previously [40], based on the method of [41]. Samples (9 g) were sonicated with 10% KOH in methanol at 70 °C for 90 min. Distilled water was added, and the samples were extracted three times with cyclohexane, evaporated under nitrogen, redissolved in methanol and analysed isocratically using a Waters Alliance HPLC system (Waters, Santa Clara, CA, USA), with methanol as the mobile phase and a flow rate of 1 mL min^−1^. Ergosterol was identified by UV detection at 282 nm. To estimate the fungal biomass, the conversion ratio of 3.8 ± 2.0-mg ergosterol per g fungal biomass was used [39].

### 2.3. DNA Extraction and Sequencing

DNA was extracted from each sandbag sample using the modified Miller method [42] with the following modification: 5 g of each freeze-dried sample were homogenised directly with glass beads and lysis buffer by vortexing at maximum speed for 10 min. DNA from soil samples was extracted using the RNeasy PowerSoil DNA Elution Kit (Qiagen, Hilden, Germany) after coextraction of RNA following the manufacturer’s instructions. The yield and purity were checked on Nanodrop and Qubit.

For the fungal community analysis, PCR amplification of the fungal ITS2 region was performed using barcoded gITS7 and ITS4 primers [43] in three PCR reactions per sample, as described previously [19]. PCR reactions contained 5 μL of 5X buffer for Q5 HiFi DNA polymerase, 5 μL of 5X Q5 HighGC Enhancer, 1.5 μL of Bovine Serum Albumin (10 mg ml^−1^), 1 μL of each primer (0.01 mM), 0.5 μL of PCR Nucleotide Mix (10 mM each), 0.25 μL Q5 HiFi DNA polymerase (2 U μL^−1^) and 1 μL of template DNA. Cycling conditions were 94 °C for 5 min, 30 cycles of 94 °C for 30 s, 56 °C for 30 s, 72 °C for 30 s and a final extension at 72 °C for 7 min. The PCR products of each sample were pooled and purified using MinElute Purification Kit (Qiagen, Hilden, Germany) and then quantified on Qubit. Equimolar mixtures of amplicons were used for library preparation with the TruSeq DNA PCR-Free LT Kit (Illumina) and sequenced in-house on the Illumina MiSeq with 2 × 250 reads (MiSeq v2 Reagent Kit).

### 2.4. Data Analysis

The amplicon sequencing data were processed using SEED 2.1.05 [44]. Briefly, pair-end reads were merged using fastq-join [45]. The ITS2 region was extracted from joined amplicons using ITS Extractor 1.0.11 [46] before processing. Chimeric sequences were detected using Usearch 8.1.1861 [47] and deleted; then, the remaining sequences were clustered at a 97% similarity level using UPARSE implemented within Usearch [48]. The most abundant sequence was selected for each cluster (operational taxonomic unit, OTU), and the closest hits at the species level were identified using BLASTn against UNITE [49] and GenBank. Where the best hit showed lower similarity than 97% with 95% coverage, the best genus-level hit was identified. The species-level analyses were performed on a dataset where OTUs belonging to the same species were combined, which reflects the fact that several fungal species have multiple dissimilar ITS sequences [19]. All other OTUs were combined into the genus of the best hit and designated “sp.”. Sequences that were identified as nonfungal were discarded. Categorisation into fungal ecological guilds was based on published literature, definitions of the groups being the same as in [50]; in addition, yeasts and moulds were separated from other saprotrophs. The moulds were defined as R-strategist fungi that are not yeasts and belong to the orders Eurotiales, Hypocreales, Mortierellales, Mucorales, Tremellales and Sporidiales [51]. The sequence data were deposited at NCBI Short Read Archive under the bio-project number PRJNA664991.

Nonmetric multidimensional scaling (NMDS) on relative abundances of fungal taxa was used with Bray-Curtis dissimilarity metrics to visualise the differences among fungal community composition in treatments. ANOVA was used to compare the ergosterol content in the ingrowth sandbags among the seasons. The effect of season on the relative abundances of fungal taxa and eco-physiological groups was analysed using Permutational Analysis of Variance (PERMANOVA). Mann-Whitney test was used to analyse the differences in relative abundances of individual taxa among seasons. All statistical analyses were performed using the R environment for statistical computing [52], and *p*-value < 0.05 was set as the limit for statistical significance.

## 3. Results

**Figure 1:**
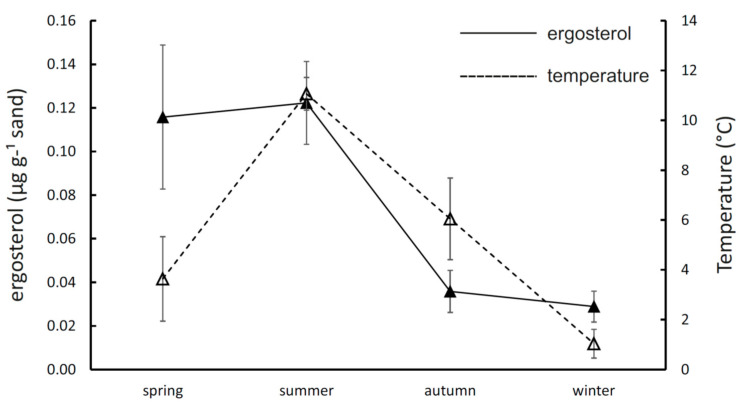
Seasonality of mycelial production and temperature in coniferous forest soil. The results indicate the mean (±SE) content of ergosterol in sandbags after incubation in situ for three months and mean (±SE) daily temperature in each season.

The mean seasonal soil temperature at a 5-cm depth ranged from 1.0 ± 1.3 °C in the winter to 11.1 ± 1.5 °C in the summer, being similar in the spring and autumn (3.6 ± 3.8 °C and 6.0 ± 3.5 °C, respectively; Figure 1). The fungal mycelial biomass measured as the ergosterol content in incubated ingrowth sandbags differed significantly among the four seasons, being threefold to fourfold higher in the spring and summer (0.116 ± 0.033 µg g^−1^ sand and 0.122 ± 0.019 µg g^−1^ sand) than in the autumn and winter (0.036 ± 0.010 µg g^−1^ sand and 0.029 ± 0.007 µg g^−1^ sand; Figure 1).

**Figure 2:**
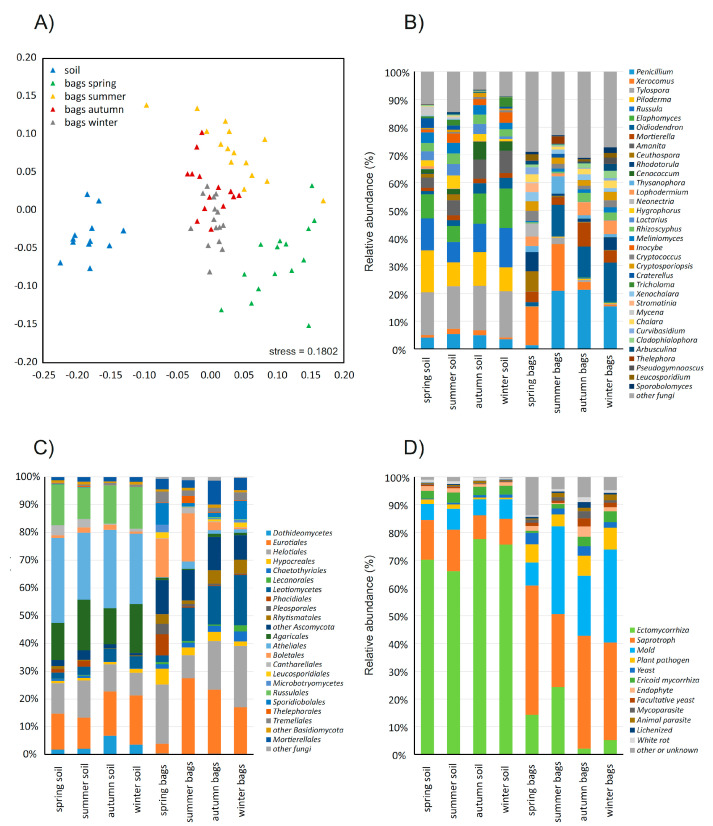
Composition of the fungal communities in temperate coniferous forest soil and in sandbags after three months of incubation in situ. Nonmetric multidimensional scaling of the fungal community composition based on Bray-Curtis dissimilarity; each point represents one sample (**A**). Composition of the fungal community at the genus level (B) and at the order level (**C**) and the share of fungal sequences grouped by ecological guilds (**D**).

The composition of the fungal community in the studied soil did not show significant differences among the seasons, while the composition of the fungal communities in the sandbags was strongly seasonal (PERMANOVA on Bray-Curtis dissimilarities, *p* < 0.001). Furthermore, the fungal communities in the sandbags were also significantly different from those in the soil (Figure 2A). In general, the sandbag communities were dominated in all seasons by fungi belonging to *Ascomycota* (63–79%), while the soil communities were dominated by *Basidiomycota* (58–64%, Figure 2B,C). The sandbag communities also had significantly higher proportions of sequences of *Mucoromycota* (from 3.7% in the summer to 9.9% in autumn) compared to soil (1.2–1.7%, Figure 2C). While the soil communities were dominated by *Tylospora* (15–17%), *Piloderma* (8–15%), *Russula* (7–14%) and *Elaphomomyces* (6–14%) species, the sandbag communities were dominated by *Penicillium* (1.5–21%), *Xerocomus* (0.6–16%) and *Oidiodendron* (1.5–14%) species, with many taxa dominant in the soil virtually missing in the sandbags (Figure 2B). This was, for example, the case of the ectomycorrhizal fungi *Tylospora fibrillosa*, *Piloderma* sp. and *Russula ochroleuca*.

**Figure 3:**
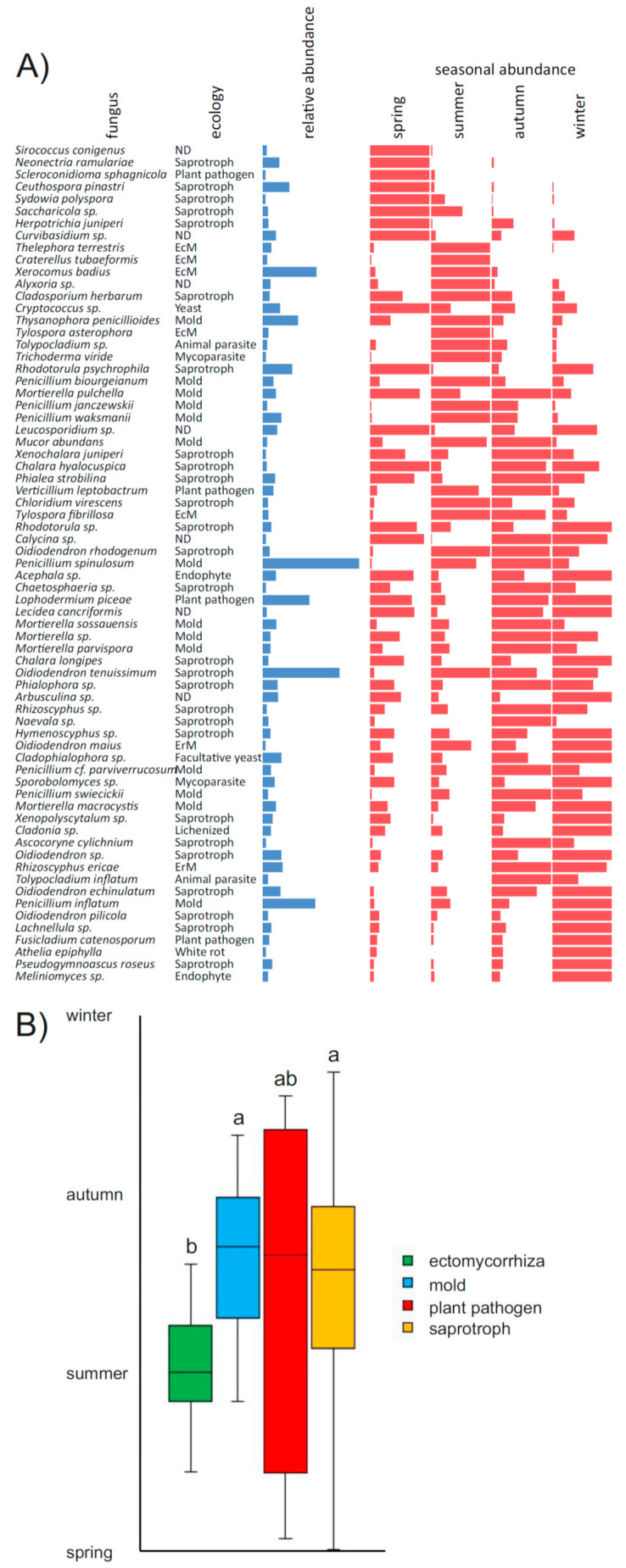
Seasonality of fungal ingrowth into the sandbags incubated seasonally in temperate coniferous forest soil. Overview of fungi with significant differences in seasonal ingrowth into in situ incubated sandbags based on the relative abundance of their sequences in the seasons (**A**); relative abundance represents the mean relative abundance of sequences across all sandbags (min. = 0.2%); seasonal abundances are represented in comparison to the season with a maximal relative abundance of sequences. Only species with significant differences in seasonal abundance (*p* < 0.05) are shown. Mean time of mycelial production for fungi of different ecological guilds (**B**). Mean time of mycelial production from the spring to winter was calculated as a timepoint with one-half of the species occurrences before and after that date. Different letters indicate significant difference (*p* < 0.05) in the time of production between ecological guilds. EcM—ectomycorrhizal, ErM—ericoid mycorrhizal and ND—not determined.

Within sandbags, as many as 69 out of 85 fungal taxa with the mean sequence abundance above 0.2% showed significant differences in relative abundance among the seasons (Figure 3A), resulting in a seasonally changing species representation in the sandbags (Figure 2B). Among the most abundant species, for example, the ectomycorrhizal *Xerocomus badius* showed the highest relative abundance in the summer, *Xerocomus pruinatus* was most common in the spring, the mould *Penicillium spinulosum* was most abundant in the summer and autumn and the *Penicillium inflatum* abundance peaked in the winter. The ericoid mycorrhizal species *Rhizoscyphus ericae* and *Oidiodendron maius* had higher abundances in the autumn and winter than in the spring and summer (Figure 3A).

Considering the representation of the ecological guilds of fungi, the soil was dominated by sequences of ectomycorrhizal fungi (66–78%), while ericoid mycorrhiza accounted for approximately 3% in all seasons, and 14–22% of the community was composed of saprotrophs and moulds (Figure 2D). The fungal community in the sandbags was composed mostly of saprotrophs (37%) and moulds (24%), followed by ectomycorrhizal fungi (11%), while the share of ericoid mycorrhizal fungi was slightly lower than in the soil (2.4%). Interestingly, the sequences of the plant pathogens were highly enriched in sandbags compared to the soil (6.5% and 1.1%, respectively), and the same was true for the yeasts (2.9% and 0.7%; Figure 2D). Unlike in the soil, the representation of certain ecological guilds in the sandbags was highly seasonal. This was most prominently seen in ectomycorrhizal fungi that represented as many as 14% of all sequences in the spring and 24% in the summer, while their share was as low as 2% in autumn and 5% in winter. This pattern was notably different from ericoid mycorrhizal fungi that were rare in the spring and summer (0.8% and 1.5%) and abundant in autumn and winter (3.4% and 3.9%). The moulds were significantly less-abundant in the spring than in the other seasons. Plant pathogenic fungi and yeasts were found in the sandbags at similar rates across all seasons (Figure 2D). When looking at the individual fungal taxa, ectomycorrhizal species tended to dominate in the sandbag community earlier in the year than saprotrophs and moulds, indicating significantly different temporal patterns of mycelial production (Figure 3). In contrast, plant pathogenic fungi were highly diverse, some of them preferably colonising the sandbags early in the seasons, while others the late (Figure 3).

## 4. Discussion

Given that ectomycorrhizal fungi account for a large proportion of the total soil microbial biomass in some systems, e.g., boreal and temperate forests [10], the death and turnover of mycorrhizal fungal tissues undoubtedly substantially contribute to the pool of dead microbial biomass that serves both as a primary resource for decomposers [6,53] and as a source of soil organic matter formation and C stabilisation [54,55]. The carbon supply to ectomycorrhizal fungi in temperate and boreal forests is restricted to certain periods of the year and, even then, varies considerably, which is accompanied by the changes of expression of ectomycorrhizal fungi, as well as all fungal transcriptions across seasons [19,34]. Similar variation is to be expected in the production of ectomycorrhizal mycelia that should reflect the C allocation of trees belowground.

Unfortunately, the biomass production was not yet studied systematically across the whole year, and our knowledge of ECM fungal biomass production is largely restricted to the period of vegetation activity. Here, we report the seasonal production of mycelia in sandbags to peak during the spring and summer (Figure 1). The summer biomass production was comparable to other studies performed in coniferous forests during the vegetation season [30,31,56] for similar timescales; however, the previous reports from boreal forests reported much faster biomass production in the late-summer and autumn [30,31]. For example, sandbag colonisation in a coniferous forest in South Sweden was fivefold higher in July–September compared to May–July, reflecting the peak of tree photosynthetic production [30]. The very low biomass production in the period of September–December that we recorded is striking, considering the fact that this autumn period is warmer than the spring, and coniferous trees are photosynthetically active for most of the time. The sandbag colonisation in the autumn was less than 25% compared to the peak in the summer and comparable to the colonisation in the following winter period when trees are inactive (Figure 1). Our results show that, despite the lower share of fungi on the total microbial transcription in the soil [19], fungi are not only metabolically active [34] but also growing, as indicated by the ergosterol accumulation in the sandbags during the cold seasons.

The use of ingrowth bags filled with sand was once proposed as a method to estimate the growth of ectomycorrhizal fungi [30], and it is still perceived as one of the primary methods of choice [14]. Indeed, the bags filled with sand reduce the ingrowth of nonmycorrhizal fungi compared to bags with sterile soil [57] and after the arrival of molecular methods that made it possible to analyse fungal communities inside sandbags, the results often confirmed that ectomycorrhizal fungi represent the majority of sandbag colonisers during the vegetation season [57,58,59]. Other reports, however, noticed the presence of other fungal guilds as well, indicating that the sandbags might reflect more the total fungal community in the studied soil than the ectomycorrhizal community only [31,56,60,61,62]. In our experimental system, we recorded a much smaller proportion of the ectomycorrhizal fungi on the total sandbag community compared to soil, especially in the winter months (Figure 3D). In addition, several dominant ECM fungi virtually failed to colonise the sandbags (Figure 3B). It should be noted that mycorrhizal taxa vary widely in their production of extraradical hyphae and, thus, the extent of mycelia in bulk soil [63]. In addition, the differential affinity of ECM fungal taxa to sandbags was occasionally observed before. Some species avoid growing in mineral substrates (*Cortinarius*), because they are adapted to an environment where they utilise organic nutrients from soil organic matter [8]. Therefore, despite being abundant on root tips, species within this genus may avoid sand-filled mesh bags, even when they are common on the root tips, while the opposite situation is the case for other taxa, e.g., *Xerocomus* spp. [64,65]). The ECM fungal community in mesh bags may, thus, not represent the community that prevails in the soil, which may be a problem in some studies.

On the other hand, the important advantage with the sandbag method is that the fungi present are recently formed, while the fungi that we can detect in the soil can be old and inactive. Despite the much lower fungal activity, the ergosterol content in the coniferous forest that we explored was almost constant between the summer and winter [19], although the rate of sandbag colonisation was approximately fourfold. There are no reliable reports on mycelial turnover rates that can put this observation into a broader context, but it is clear that fungi differ in the share of active and inactive biomass that they produce and sustain [5]. Additionally, when comparing the total soil community and the sandbag subcommunity, it should be always considered that the former represents the static composition of the standing mycelia, while the latter stands for the recently active fraction.

Whatever imperfect may be the representation of the total fungal community in the sandbags, their use allows to assess the seasonality of mycelial production across those taxa that colonise them. In this respect, our results show that the seasonal variation in mycelial growth is widespread among fungi, since more than 80% of dominant fungi, belonging to a wide range of ecological guilds, show preferential sandbag colonisation in certain seasons (Figure 3A). In a previous study, we demonstrated seasonal differences in the gene transcription of soil bacteria from the same ecosystem and shown that their growth varies across seasons. While the opportunistic bacteria, in contrast to decomposers of complex biopolymers, were found to proliferate rapidly in the summer when rhizodeposition is high and reduce their growth in the winter, the decomposers grew at similar rates in both seasons [33]. In analogy, we would expect the share of the mycelial production of similar opportunistic fungi—moulds and ECM fungi that obtain carbon from their hosts—to increase in sandbags in the vegetation season, while other saprotrophs are more likely to be represented in the winter. Our results indeed show that sandbag colonisation by ECM is higher in the spring and summer and low in the autumn and winter (Figure 2D). We also demonstrate that ECM fungi grow during the whole year, irrespective of the activity of their hosts. However, if we consider the ergosterol content as a proxy of the biomass and the relative abundance of sequences as the representation of ECM in the fungal community, the sandbag colonisation in the spring and summer might account for some 0.0160–0.0300 µg ergosterol g^−1^ sand and only 0.0007 and 0.0015 µg g^−1^ sand in the autumn and winter, respectively, i.e., with an approximate mycelial production of 2–5% of peak productivity in the summer. It is unclear why the share of ECM is lowest in the autumn when temperatures are still favourable for tree photosynthetic production and *Picea abies* root tip production is similar as in the spring [66].

Compared to ECM fungi, the relative abundance of ericoid mycorrhizal fungi in the sandbags was higher in the autumn and winter, although their host plants were, most of the time, inactive under the snow cover. Previously, the ericoid mycorrhizal fungal gene repertoire revealed a capacity for a dual saprotrophic and biotrophic lifestyle [67]. Indeed, the results of our study provide strong evidence that the dependence of ericoid mycorrhizal fungi on their host plants is weaker compared to ectomycorrhizal fungi and that ericoid mycorrhizal fungi are able to proliferate in soil even if their host plants are not active.

Contrary to our expectations, the ratio of moulds and saprotrophs was higher in the autumn and winter than during the months of tree production. High shares of moulds in the winter were also reported previously for soils [68]. Since moulds, such as *Mortierella* spp., frequently colonise decomposing fungal mycelia [7,53], their high abundance in the winter may indicate their activity connected with the decomposition of seasonally produced mycelia of the ECM fungi. Interestingly, plant pathogens and unicellular yeasts were enriched in the bags across all seasons. While, for the former group, it makes sense to explore the substrate and search for host roots, the appearance of yeasts in the sandbags is more difficult to explain. Yeasts, unlike filamentous fungi, are immobile and can enter sandbags only passively, such as with water flow. Moreover, many of them seem to prefer easily decomposable substrates [69] that are largely absent in the sandbags.

Our results also indicate that many fungi produce most mycelia at certain period of time during the year. This is an interesting and unique observation that would deserve further exploration.

A few studies previously reported on the seasonality of the biomass production of individual ectomycorrhizal species, typically those picked for revenue, e.g., *Boletus edulis*, showing the highest biomass in February, *Lactarius deliciosus* in December [70] or *Lactarius vinosus* in February and May [71], in all cases primarily reflecting higher moisture contents in the seasonally dry forests that were studied. Among the species explored here, the ectomycorrhizal taxa consistently proliferated, preferably in the spring and summer, which is in-line with the initial and peak activity of their tree hosts.

## 5. Conclusions

Our results indicate that fungal biomass production peaks in the summer and spring but also suggests that fungi, including ectomycorrhizal root symbionts, keep proliferating also during the winter under snow cover. It remains unclear why the autumn season with favourable temperatures and tree activity is associated with decreased growth. Since much less seasonality is seen in the composition of the total soil community, it appears that the replacement of old mycelia by various fungi takes place at different seasons. Our results also clearly demonstrate that fungal taxa differ in their seasonal patterns of mycelial production. In the future, it would be advisable to develop tools to study the biomass turnover of all ectomycorrhizal species, including those that avoid sandbags, and to advance our knowledge of the seasonality of fungi in soils.
